# Microscopic Detection of Intestinal *Sarcocystis* Infection Diagnosed in International Travelers at the Institute of Tropical Medicine, Antwerp, Belgium, from 2001 to 2020

**DOI:** 10.4269/ajtmh.22-0577

**Published:** 2023-06-05

**Authors:** Steven Van Den Broucke, Pierre Dorny, Marjan Van Esbroeck, Emmanuel Bottieau

**Affiliations:** ^1^ Department of Clinical Sciences, Institute of Tropical Medicine, Antwerp, Belgium;; ^2^ Medical Helminthology, Department of Biomedical Sciences, Institute of Tropical Medicine, Antwerp, Belgium

## Abstract

Although a stay in tropical regions is considered a risk factor for acquiring *Sarcocystis* infection, to date intestinal sarcocystosis has never been described in returning travelers. We did a retrospective cross-sectional study, retrieving all *Sarcocystis* spp. microscopy-positive stool results of individuals who attended the travel clinic of the Institute of Tropical Medicine, Antwerp in the period from 2001 to 2020. We reviewed the medical records and report on the epidemiology and clinical features of intestinal sarcocystosis in international travelers. In 57 (0.09%) of 60,006 stool samples, oocysts or sporocysts of *Sarcocystis* spp. were found, often together with other intestinal infections. Twenty-two (37%) individuals were asymptomatic, 17 (30%) had intestinal ± extraintestinal symptoms, and 18 (32%) had extraintestinal symptoms only. Only one traveler had symptoms suggestive of acute gastrointestinal sarcocystosis without an alternative diagnosis. Intestinal *Sarcocystis* infection predominated in male travelers. At least 10 travelers most likely acquired intestinal *Sarcocystis* in Africa, where it was never described before. In a national reference travel clinic in Europe, the presence of intestinal *Sarcocystis* oocysts is a rare finding, predominant in male travelers. Infection with this parasite infrequently leads to suggestive clinical manifestations such as acute gastrointestinal symptoms. Our data strongly suggest that *Sarcocystis* can be acquired throughout tropical areas, including Africa.

## INTRODUCTION

Sarcocystosis, caused by protozoa of the apicomplexan genus *Sarcocystis*, is a worldwide zoonotic infection with more than 200 known species. Most *Sarcocystis* species have a species-specific obligate two-host life cycle: an intermediate or prey host and a definitive or predator host. After consumption of undercooked meat, the sexual reproduction of *Sarcocystis* occurs in the small intestinal epithelium of a definite (predator) host, which excretes oocysts or sporocysts in the stools. When the intermediate (prey) host ingests the sporocysts, sporozoites are released and undergo asexual multiplication in the endothelial cells of small arteries, including in the intestines, releasing merozoites. In muscle cells, merozoites develop into sarcocysts containing infective bradyzoites that are ingested by the definitive host. Humans are the natural definitive or accidental intermediate hosts of several *Sarcocystis* spp.^1^ *Sarcocystis hominis* and *Sarcocystis suihominis* cause intestinal sarcocystosis acquired from eating undercooked meat, with humans serving as the definitive host and cattle and pigs as intermediate hosts, respectively.

The prepatent period of *Sarcocystis* spp. in humans, namely the time from infection to the appearance of oocysts or sporocysts in the stool, varies from 9 to 18 days, after which oocysts or sporocysts are usually excreted up to 6 weeks, with rare extreme patent periods described up to > 120 days in an experimentally infected person.^1^^–^^3^ The incubation period, namely the time from infection to the appearance of symptoms, varies from 3 hours to 1 week. It is assumed that most natural human intestinal sarcocystosis cases go unrecognized,^4^^,^^5^ even though symptoms are reported in 10% of infected individuals,^6^ including severe and fatal enteritis.^7^ The range of the clinical manifestations depends on the intensity of the infection and the *Sarcocystis* species. *Sarcocystis suihominis* is believed to cause more severe symptoms than *S. hominis*.^8^^,^^9^ Experimental infections of healthy volunteers have led to a spectrum of symptoms ranging from asymptomatic to severe gastroenteritis and eosinophilia, combined with extraintestinal symptoms (low-grade fever) in rare occasions.^1^^,^^2^ If symptoms occur, they are usually self-limiting and often resolve within 36 hours, but cases of prolonged diarrhea up to 28 days have been reported.^2^^,^^10^ Humans can develop muscular sarcocystosis as an accidental, dead-end intermediate host for *Sarcocystis nesbitti*, a species in Malaysia for which snakes are the definitive hosts and nonhuman primates are the natural intermediate hosts.^1^^,^^2^^,^^11^

Traveling to tropical regions is considered a risk factor for acquiring *Sarcocystis* infection, and about 100 clustered cases of muscular sarcocystosis were reported after a stay on Tioman Island (Malaysia) in 2011 and 2012.^12^^,^^13^ To date, intestinal sarcocystosis has never been studied in returning travelers. The Institute of Tropical Medicine, Antwerp (ITMA) hosts the national reference laboratory for diagnosing tropical infectious diseases in Belgium, with on average 6,500 consultations a year for posttravel care. A substantial proportion of patients at ITMA present with gastrointestinal symptoms and/or wish to rule out the presence of parasites acquired abroad, and undergo a specialized diagnostic workup including stool investigation for parasites. Consequently, in a proportion of our patients, *Sarcocystis* oocysts or sporocysts are found. The present study aims to assess the frequency, epidemiology, and presentation of intestinal *Sarcocystis* spp. infections in travelers during a 20-year period at ITMA.

## MATERIALS AND METHODS

### Study setting and population.

For this retrospective cross-sectional study, data retrieval was performed on the Laboratory Information System (LAB400; Cegeka, Hasselt, Belgium) of ITMA’s medical laboratory using BusinessObjects (SAP, Paris, France) to extract all *Sarcocystis* spp. microscopy-positive stool results of individuals who attended the travel clinic of ITMA in the period from 2001 to 2020. We reviewed the medical records of all travelers with the presence of *Sarcocystis* spp. in their stools during this period.

### Laboratory workup.

Stool samples were subjected to microscopic examination of direct smears and wet mounts after formalin-ether concentration.^14^ As of 2010, part of the stools was in addition mixed with a sodium acetate-acetic acid-formalin (SAF) solution within 20 minutes and examined by microscopy after iron-hematoxylin Kinyoun staining, if requested by the treating physician. In case a stool sample could not be produced at the time of the consultation at ITMA, the patient received a package to collect stools at home and instructions to mix part of the stools immediately with SAF solution, and send both fixed and unfixed portions to ITMA.

### Statistical analysis.

For all cases of intestinal *Sarcocystis* infection, relevant clinical data were extracted, deidentified, and entered into a Microsoft Excel sheet. Variables included demographic data, most recent travel destination, duration of travel, type of traveler, timing of stool exam after the return date, time of consultation after travel, risk factors for exposure, clinical symptoms with a detailed assessment for the presence of diarrhea, abdominal pain, nausea or vomiting, fever, extraintestinal symptoms, coinfections, and other diagnoses, treatment administered, and presence of *Sarcocystis* in a follow-up stool sample (if done).

**Figure 1.**
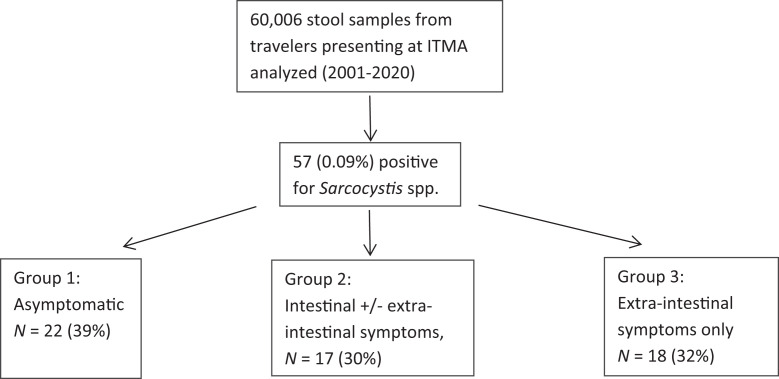
Flowchart and distribution of travelers.

Next, we categorized all reviewed *Sarcocystis*-positive cases in three groups according to the clinical presentation: 1) asymptomatic, 2) intestinal symptoms ± extraintestinal symptoms, and 3) extraintestinal complaints only (Figure 1).

### Case management.

Given the lack of clinical trial data, no formal guidelines for the treatment of intestinal sarcocystosis exist. Infections are often asymptomatic or self-limiting and treatment is usually not required. Anticoccidial agents such as cotrimoxazole or albendazole have been used but their efficacy remains to be demonstrated.^11^ When patients were symptomatic and no alternative diagnosis was found, cotrimoxazole or albendazole was prescribed.

### Ethics.

This was a retrospective analysis of deidentified data collected during routine clinical care over 20 years. Laboratory and clinical data were retrieved from medical files via an encoded link and were deidentified for analysis according to Belgian legislation and EU General Data Protection Regulation. No written informed consent had been obtained from individual participants, but data are fully deidentified. Additionally, a presumed consent (opt-out) procedure has been in place since March 2008 at ITMA that covers use in secondary research of deidentified clinical and laboratory data.

## RESULTS AND DISCUSSION

### Epidemiological features of intestinal *Sarcocystis* infection.

From January 2001 to December 2020, 60,006 stool samples of patients presenting at the ITMA travel clinic were tested. In 60 (0.09%) of these stool samples, oocysts or sporocysts of *Sarcocystis* spp. were detected, of which three patients had not traveled and were excluded from our analysis. Diagnosis was done in 53 samples after formalin-ether concentration, in 4 samples in the direct smear, in 1 sample in SAF-fixed stools after iron-hematoxylin Kinyoun staining, and in 2 samples in both the direct smear and after concentration. The clinical presentation of the remaining 57 travelers is described in detail in Table 1. Cases were not evenly distributed throughout the study period, with 51 cases (89%) of *Sarcocystis* found from 2001 to 2014 and 6 (11%) found from 2016 to 2020 (there were no cases in 2015). The average yearly number of stools analyzed from 2001 to 2014 was 3,406, whereas it was 2,005 for the period 2016–2020, with only 870 stool samples examined in 2020 because of the COVID-19 pandemic. More *Sarcocystis* infections were detected during summer months (data not shown) but the same increase is seen with *Blastocystis* and *Giardia* because more travelers consult at ITMA during summer. Fifty-four infected individuals were born in Belgium, two travelers originated from the Democratic Republic of Congo, and one was from France. The mean age was 41 years (range 0–76 years) with a male (37/57) to female (20/57) ratio of 1.85, whereas this ratio was 1.21 for the 60,006 stool samples analyzed. Regions of most recent travels (and presumed exposure) were Africa in 44 travelers (77%), South Asia and Southeast Asia in 5 (9%), Central America and South America in 6 (11%), and Europe in 2 (3%). For 43 of 57 patients, the time of consultation after travel was known. *Sarcocystis* infection was most likely acquired abroad in 10, 0, 1, and 0 travelers from Africa, Asia, America, and Europe, respectively (interval return date to consultation at ITMA < 9 days and duration of travel > 6 weeks, which makes infection before or after travel less likely) (Table 1). Fourteen travelers most likely acquired *Sarcocystis* in Belgium because the time of travel plus the time after return and presentation at ITMA was less than 9 days, indicating that acquisition was pretravel, or they presented 2 months or later after returning, which suggested posttravel acquisition (prepatent period maximum 18 days + oocyst/sporocyst shedding is usually not more than 6 weeks). Data on exposure to raw meat were hardly available in patient files, and were explicitly mentioned for four travelers only.

**Table 1:** Clinical presentation of travelers with intestinal *Sarcocystis* infection

Patient characteristics (*N* = 57)	
Age	41 years (mean), range 0–76 years
Male	37 (65%)
Female	20 (35%)
Type of travel	
Expat (> 6-month residence)	32 (56%)
Permanently living abroad	1 (2%)
Tourism	9 (16%)
Business	8 (14%)
Visiting friends and relatives	1 (2%)
Mariner	1 (2%)
Unknown	5 (9%)
Region of travel	
Africa	44 (77%)
Asia	5 (9%)
Central America	6 (11%)
Europe	2 (3%)
Time between return date and consultation known	43/57 (75%)
*Sarcocystis* most likely acquired abroad*	11/43 (26%)
Africa	10/31
Asia	0/4
Central America	1/6
Europe	0/2
*Sarcocystis* possibly acquired abroad†	18/43 (42%)
Africa	10/31
Asia	4/4
Central America	3/6
Europe	1/2
*Sarcocystis* unlikely acquired abroad‡	14/43 (33%)
Africa	11/31
Asia	0/4
Central America	2/6
Europe	1/2
Patients with any symptom	35/57 (61%)
Symptoms present before travel	5/35
Symptoms started during travel	10/35
Symptoms started ≤ 1 week after travel	4/35
Symptoms started > 1 week after travel	4/35
Symptom onset not known	11/35

* Interval return date to consultation at the Institute of Tropical Medicine, Antwerp (ITMA) < 9 days (= minimal prepatent period [= time from infection to egg shedding]) and duration of travel > 6 weeks (6 weeks is considered the maximal egg excretion period in natural infection).

† Interval return date to consultation at ITMA > 9 days and/or duration of travel < 6 weeks.

‡ Interval return date to consultation at ITMA > 2 months (maximal prepatent period = 18 days + 6 weeks maximal egg excretion period in natural infection) or duration of travel + time until return to consultation at ITMA < 9 days (= shorter than the minimal prepatent period).

### Clinical features of intestinal *Sarcocystis* infection.

Of the 57 travelers, 22 (39%) were asymptomatic at the time of diagnosis, 17 (30%) had intestinal symptoms associated or not with extraintestinal symptoms, and 18 (32%) had extraintestinal symptoms only (Figure 1). Of the 17 travelers with intestinal symptoms, 14 had extraintestinal symptoms (32 in total with extraintestinal symptoms). Symptoms were acute (≤ 3 weeks) in 6 patients (3 with intestinal symptoms and 3 with extraintestinal symptoms only) and chronic (> 3 weeks) or recurrent in 18 (11 with intestinal symptoms and 7 with extraintestinal symptoms only). Data on symptom duration were lacking in 10 (7 in the extraintestinal symptoms-only group) (Supplemental Tables 1–3).

Intestinal symptoms were abdominal pain (*N* = 12; data were lacking in 1 individual), diarrhea (*N* = 10), and nausea or vomiting (*N* = 5; data were lacking in 1 individual). Fever was the most common extraintestinal symptom (*N* = 7); other extraintestinal symptoms varied widely. In 32 of 57 (56%) of our travelers, 55 concurrent diagnoses (of which 51/55 [93%] were pathogenic and nonpathogenic infections) were found. Details of the clinical presentation and cofindings are presented in Supplemental Tables 1–3.

In none of the 18 patients with extraintestinal symptoms only were symptoms likely attributable to intestinal *Sarcocystis* infection. In 9 of the 17 patients presenting with intestinal symptoms, an alternative diagnosis more likely to explain the symptoms was found (of note: we considered *Blastocystis hominis*,* Entamoeba dispar*, *Entamoeba coli*,* Entamoeba hartmanni*, and *Endolimax nana* as nonpathogenic). Of the 8 patients with intestinal symptoms without an alternative diagnosis, symptoms were acute (< 3 weeks) in 1 and chronic (> 3 weeks) in 6, and data on symptom duration were lacking in 1. Thus, only one patient presented with acute intestinal symptoms—suggestive of intestinal sarcocystosis—without an alternative diagnosis. He came back from a 4-day trip to Ghana, Togo, and Benin and presented with a 3-day history of diarrhea, abdominal pain, nausea, headache, and fatigue.

Treatment was proposed to 4 of the 22 asymptomatic patients (2 in case abdominal symptoms would occur), and was given in 11 of the 17 patients with intestinal symptoms and in 9 of the patients with extraintestinal symptoms. In nine patients a follow-up stool sample was available (after 1 week [*N* = 4], 1 month [*N* = 2], 3 months [*N* = 1], and 1 year [*N* = 2]) and in none was *Sarcocystis* detected.

Our study has several limitations. The retrospective nature of the study with the lack of systematic data collection warrants cautious conclusions. Stool samples were not obtained in all travelers, and therefore an unknown number of *Sarcocystis* infections have likely been missed. Fecal shedding of *Sarcocystis* oocysts/sporocysts is usually light and thus may easily be overlooked, translating into a lower sensitivity of direct microscopy compared with novel molecular methods.^15^ Consequently, even though all examined stool specimens underwent formalin-ether concentration, which increases the sensitivity, our numbers might underestimate the actual prevalence in international travelers. Correct diagnosis of intestinal sarcocystosis is notoriously difficult because the incubation period (3 hours to 1 week) is shorter than the prepatent period (9 to 18 days); hence, oocysts may not yet be shed in feces when symptoms are present. This may have led to an underestimation of symptomatic sarcocystosis in our cohort. The lack of anamnestic details on the consumption of raw or undercooked meat makes assigning symptoms to *Sarcocystis* problematic. The number of *Sarcocystis* infections was not evenly distributed during the study period and, during the last 6 years of the study, there was a decreasing tendency to screen stool samples for parasites in asymptomatic travelers. We cannot make firm statements regarding prevalence according to geographic region because a relatively high number of cases detected in travelers returning from Africa most likely reflects the higher proportion of travelers from this continent at ITMA rather than a truly higher prevalence. Despite the above limitations, our study describes one of the most extensive series of intestinal *Sarcocystis* infections and is the first study that reports on fecal microscopy-positive sarcocystosis in returning travelers.

Intestinal *Sarcocystis* infections in humans have been reported worldwide, with a prevalence of mostly community-based studies between 1.1 and 10.4% in Europe, between 0.4 and 23.2% in Asia, 0.5% in Australia, and 0% in Argentina.^2^ Importantly, cases of intestinal *Sarcocystis* infection have never been reported in Africa and the Middle East. The most recent travel destination in our cohort was Africa, with 44 travelers. We found at least 10 travelers who most likely acquired intestinal *Sarcocystis* in an African country (duration of travel > 6 weeks = maximal shedding period and consultation at ITMA < 9 days after travel [= minimal prepatent period]). Likewise, 14 of our travelers presented > 2 months after their return date and probably acquired intestinal sarcocystosis in Belgium. The detection of *Sarcocystis* spp. in stools of three patients who had not traveled indeed supports the possibility of acquiring *Sarcocystis* in Belgium. A recent study detected *S. hominis* in 21% of bovine carcasses from a Belgian abattoir, underscoring potential food safety issues.^16^ Of note, in the nine individuals for whom a follow-up sample was available, in none of them was *Sarcocystis* found. This is in line with the knowledge that the excretion of oocysts is usually transient and short lived.

Another striking finding was the predominance of *Sarcocystis* infection in male travelers. A study in two countryside villages in Guangxi found 22 men and 5 women out of 501 persons examined were positive for *S. suihominis* stool infection.^17^ Another study in 362 Thai laborers found 70 out of 246 male laborers infected with *Sarcocystis* versus 14 out of 116 female laborers, which the authors attributed to a larger number of male laborers traveling abroad.^4^ In our cohort, the male/female ratio was 1.85 versus 1.21 in all stool samples examined during the study period, suggesting that traveling does not explain the male predominance and that other factors, such as higher meat consumption in men, might influence the risk of acquiring *Sarcocystis* infection.^18^

Our study highlights that it is challenging to determine whether symptoms should be assigned to *Sarcocystis* or whether this infection should be considered an innocent bystander phenomenon. Gastrointestinal symptoms appearing shortly after consumption of raw or undercooked meat lead to a presumptive diagnosis of intestinal sarcocystosis, which is confirmed with the identification of *Sarcocystis* spp. oocysts/sporocysts in the traveler’s feces starting a minimum of 9 days after the meat consumption.^2^^,^^19^ Because of the retrospective character of the study, in only four of our travelers, anamnesis revealed consumption of raw meat with the interval to symptoms specified in two, whereas this information was lacking in the other travelers. This stresses the importance of a comprehensive food anamnesis in returning travelers, because they are at risk for other meat-borne diseases such as those caused by *E. coli*,* Salmonella*,* Shigella*,* Campylobacter*,* Listeria monocytogenes*,* Brucella*,* Trichinella* spp., *Taenia* spp., *Toxoplasma gondii*,* Mycobacterium bovis*, and toxins produced by *Staphylococcus aureus*,* Clostridium* species, and *Bacillus cereus*.^20^ Identification of *Sarcocystis* in stool specimens should warrant clinicians to pursue a detailed record of raw food consumption. Because infection with *Sarcocystis* spp. can be prevented by thorough cooking or freezing of meat that kills bradyzoites in the tissue cysts, this study again highlights the importance of safe food intake during pretravel counseling.

Our findings confirm the largely asymptomatic nature of intestinal *Sarcocystis* infection: of the 57 diagnosed *Sarcocystis* infections, only one patient presented with acute intestinal symptoms without a more likely alternative diagnosis. We believe that alternative diagnoses such as irritable bowel syndrome, for which Travelers’ diarrhea is a known risk factor,^21^ are likely at stake in cases of chronic (> 3 weeks) presentation, although we cannot formally exclude an etiological role of *Sarcocystis* infection. The same reasoning applies to patients presenting with extraintestinal symptoms only. Although we cannot formally exclude sarcocystosis as the explanation for the symptoms, at least partly, we considered this unlikely. In patients presenting with intestinal and extraintestinal symptoms, the hypothesis of a dihomoxenous life cycle could offer an explanation for the combination of intestinal and muscular sarcocystosis.^22^^,^^23^ However, for *Sarcocystis*, this is only described in giant lizards living on the Canary Islands. It is important to note that these Sarcosporidia are transmitted cannibalistically: these island-dwelling lizards have the habit of eating each other’s autotomized tails during persecution chases. So, the dihomoxenous life cycle is only possible in cases of cannibalism or a person eating parts of his own body. Therefore, we think it is improbable that a dihomoxenous life cycle in humans with an unknown *Sarcocystis* species could explain the combination of intestinal and extraintestinal symptoms.

In conclusion, in a national reference travel clinic in Europe, the presence of intestinal *Sarcocystis* is a rare finding. Intestinal sarcocystosis predominates in male travelers. In our experience, infection with this parasite infrequently leads to suggestive clinical manifestations such as acute gastrointestinal symptoms. Our data strongly suggest that *Sarcocystis* can be acquired throughout tropical areas, including Africa, where it was never described before. Our study pleads for robust multicentric prospective studies in travelers to clarify the disease burden and optimal management of *Sarcocystis* in travelers.

## Supplemental Material


Supplemental materials
