# Supplementary material for: Microscopic Detection of Intestinal Sarcocystis Infection Diagnosed in International Travelers at the Institute of Tropical Medicine, Antwerp, Belgium, from 2001 to 2020
Source: Am J Trop Med Hyg. 2023 Jun 5;109(2):327–31. doi: 10.4269/ajtmh.22-0577 (PMC10397430; doi:10.4269/ajtmh.22-0577)
Supplement: Supplementary file 1 [file tpmd220577.SD1.pdf]

Supplementary table 1: Asymptomatic travelers

Last travel destination	Other infections	Date of diagnosis	Age, year/gender	Duration of travel	Timing stool exam after return date	Treatment
Benin		30/07/2010	33/F	> 1 year	23 days after return	-
Burundi	*E. nana, E. hartmanni, E. dispar*	12/08/2010	42/M	> 1 year	3 days after return	-
Burundi	*B. hominis*	06/07/2012	39/M	> 1 year	1 day after return	CTX
Cambodia		18/10/2010	29/M	5 months	no data	-
Cameroun		25/09/2001	59/M	4 months	no data	no
DR Congo		12/08/2005	40/M	> 1 year	27 days after return	no
DR Congo		23/05/2007	32/M	1 month	3 months after return	CTX if ambdominal complaints
Egypt		23/09/2005	55/M	1 month	8 months after return	no
Ghana	*E. coli*	04/01/2013	40/M	> 1 year	2 weeks after return	-
Ivory Coast	*E. nana, E. hartmanni, E. coli*	20/05/2011	72/M	3 months	no data	-
Ivory Coast	*B. hominis, E. coli*	05/08/2013	45/M	> 1 year	no data	-
Ivory Coast	*B. hominis*	30/04/2014	46/M	> 1 year	no data	-
Kenya		22/07/2010	61/M	> 1 year	3 days after return	-
Kenya	*B. hominis, E. coli*	22/06/2011	46/M	1 year	1 day after return	CTX if ambdominal complaints
Madagascar		25/05/2007	28/M	> 1 year	no data	no
Madagascar	*T. trichiura, B. hominis*	31/07/2019	40/M	> 1 year	no data	-
Rwanda		13/07/2004	49/M	> 1 year	2 days after return	no
Rwanda		04/08/2006	43/F	> 1 year	no data	no
Rwanda	*E. nana*	09/07/2012	10/F	> 1 year	3 days after return	-
Rwanda	*B. hominis*	25/08/2020	41/M	> 1 year	no data	-
Senegal	*E. nana, E. coli*	05/08/2002	31/M	2 years	8 days after return	CTX
Tsjaad	*B. hominis, E. nana*	05/08/2016	18/M	> 1 year	no data	-

B. hominis = Blastocystis hominis, E. coli = Entamoeba coli, E. dispar = Entamoeba dispar, E. hartmanni = Entamoeba hartmanni, E. nana = Endolimax nana, T. trichiurus = Trichuris trichiura

CTX = trimethoprim-sulfamethoxazole

Supplementary table 2: Travelers with intestinal +/- extraintestinal symptoms

Last travel destination	Other infections/diagnoses	Date of diagnosis	Age, year/gender	Duration of travel	Symptom duration	Timing symptom onset after return date	Symptoms	Treatment
Burkina Faso	*Cryptosporidium ubiquitum, B. hominis, E. coli,* Lyme disease	12/08/2020	63/M	> 1 year	2 months	During stay	Fatigue, erythema migrans 5 months earlier, precordial pain towards left arm, eye irritation and redness	-
Burundi		04/07/2007	7/F	1 month	3 months	During travel	Urinary incontinence, pschychological problems, diarrhea and abdominal pain for several months	CTX
Columbia	*G. intestinalis*	20/06/2014	26/F	3 months	6 months	Last days of travel	Since Giardia infection previous travel 6 months ago stools irregular, insomnia	-
DR Congo		24/08/2004	71/F	> 1 year	several years	20 years after return	Regularly post-prandial diarrhea, urge-incontinence	CTX + primaquine
DR Congo	*G. intestinalis,* Loeffler syndrome	07/10/2014	42/M	Resident D	no data	no data	Blood in stool, soft stool, pain right fossa; 9 months earlier eosinophilia 1290, dyspnea for 10 days	
Ethiopia		12/06/2003	31/F	5 months	no data	no data	Meteorism, spasms, abdominal cramps	CTX
Ghana, Togo, Benin	*B. hominis, E. nana*	25/06/2018	39/M	4 days	3 days	Last days of travel	Diarrhea, abdominal pain, nausea, headache, fatigue	Prednisolone 5 mg 4x1/d
Namibia and South-Africa		04/08/2020	67/F	3 weeks	2 months	3 months after return	Since 2 months loose stools, left fossa pain; antibiotics for diverticulitis	-
Peru	*A. lumbricoides*	23/11/2011	30/F	> 1 year	no data	no data	Weight loss, nausea, anorexia and abdominal pain	ALB, CTX if complaints persist
Rwanda	*B. hominis, E. coli*	11/08/2005	45/M	2 years	2 months	2 months after return	Vomiting and malaise 2 months ago, since then pain stomach, anorexia, weightloss, flatulence	no
Sierra Leone	Cellulitis	27/09/2011	51/M	no data	9 months	1 day after return	Since 9 months fever 1x/2-3m, recurrent pain right flanc; since 1 week abdominal wall/pelvis sores	ALB
Suriname	*D. fragilis, B. hominis*	17/05/2010	0/F	3 months	12 days	5 days after return	Nausea, vomiting and fever	Metronidazole
Thailand	*Anisakis* serology pos.	16/05/2012	49/M	1 month	5 days	Last days of travel	Diarrhea, abdominal pain, nausea, vomiting, weight loss and feverish	Tinidazole
Tunis	*E. vermicularis, B. hominis, E. nana, E. hartmanni*	18/06/2014	48/M	1 month	chronic	Before travel	Chronique diarrhea, abdominal pain, flatulence, anorexia, intermittent fever and allergic rash, recurrent herpes labialis	Cotri 10d
Ethiopia		05/03/2012	28/M	3 weeks	2 months	Before travel	Since 2 months intermittent diarrhea, since 4 days pain neck, arm	CTX if complaints persist
Papoea New Guinea	*B. hominis*	30/03/2012	30/M	18 days	2 years	Before travel	feeling, fatigue	-
France	Lupus erythematodes	06/12/2001	44/F	1 month	12 years	Before travel	Chronic (12 years) skin rash, pain right upper quadrant	CTX

A. lumbricoides = Ascaris lumbricoides, B. hominis = Blastocystis hominis, D. fragilis = Dientamoeba fragilis, E. coli = Entamoeba coli, E. hartmanni = Entamoeba hartmanni, E. nana = Endolimax nana, E. vermicularis = Enterobius vermicularis, G. intestinalis = Giardia intestinalis

ALB = albendazole, CTX = trimethoprim-sulfamethoxazole

Supplementary table 3: Travelers with extraintestinal symptoms only

Last travel destination	Other infections / diagnoses	Date of diagnosis	Age, year / gender	Duration of travel	Symptom duration	Timing symptom onset after return date	Symptoms	Treatment
Burundi	*E. hartmanni*	22/02/2002	67/M	> 1 year	6 months	During stay	Bilateral hand paralysis, vision loss, eosinophilia	no
Caraibes		03/06/2003	68/M	1 month	3 months	Last days of stay	Stifness arms, pain hands during cruise	no
DR Congo		31/03/2004	55/M	> 1 year	no data	no data	Spot on glans penis, pain on biopsy nerve	CTX
DR Congo		20/07/2004	38/F	2 months	2 years	Last days of stay	Facial oedema since 10 days	no
DR Congo	*E. dispar, E. coli*	02/11/2005	44/F	> 1 year	no data	no data	Knee pain, bone pain, bronchitis-like symptoms	CTX
DR Congo	*B. hominis, E. coli*	21/05/2013	39/F	> 1 year	recurrent	no data	Recurrent rhinitis and otitis media	-
Ethiopia	*B. hominis*	12/06/2003	29/M	5 months	no data	no data	Athlete food	CTX
Indonesia	*B. hominis,*	12/04/2006	43/M	4 months	no data	no data	Skin spots, hemorroids	no
Ivory Coast	*E. hartmanni*	10/12/2001	55/F	> 1 year	no data	no data	Itch hands and neck, lumbal pain	no
Kenya	*G. intestinalis, B. hominis*	18/12/2008	7/F	> 1 year	recurrent	no data	Recurrent staphylococcus skin infections, itch, bleeding nostrils	-
Libya		04/01/2005	18/F	3 weeks	3 weeks	1 week after return	Lower back pain	CTX if abdominal complaints
Peru		19/07/2013	30/F	1 year	years	years after stay	Since several years allergies, skinrash, swollen joints, muscle pain	ALB
Senegal		28/12/2005	48/F	> 1 year	no data	no data	Troubles with memory, hallucinations, concentration troubles, hand and feet swollen	CTX
Uganda	*B. hominis*	27/06/2011	21/F	3 months	Several da	End of stay	Headache	-
Vietnam		17/05/2004	56/M	8 days	5 days	5 days after return	Chills, nightsweats, headache, diffuse myalgia, rash on legs, red painful knee, dark urine	CTX + azithromycin
West- and Central Africa		02/04/2004	37/M	2 months	no data	no data	Swollen hand evoced by DEC	no
Mexico		05/04/2012	75/M	2 weeks	months	Before travel	In Cameroun (before Mexico) upper airway infection, persistent fatigue	CTX
Spain		11/06/2008	53/M	1 month	8 weeks	Last day of stay	Since 8 weeks muscle cramps shoulders, arms and wrist with swelling fingers, fever 6 weeks ago	CTX

B. hominis = Blastocystis hominis, E. coli = Entamoeba coli, E. dispar = Entamoeba dispar, E. hartmanni = Entamoeba hartmanni, G. intestinalis = Giardia intestinalis
ALB = albendazole, CTX = trimethoprim-sulfamethoxazole
